# Antagonistic activity of endophytic actinobacteria from native potatoes (*Solanum tuberosum* subsp. *tuberosum* L.) against *Pectobacterium carotovorum* subsp. *carotovorum* and *Pectobacterium atrosepticum*

**DOI:** 10.1186/s12866-021-02393-x

**Published:** 2021-12-07

**Authors:** Natalia Padilla-Gálvez, Paola Luengo-Uribe, Sandra Mancilla, Amandine Maurin, Claudia Torres, Pamela Ruiz, Andrés France, Ivette Acuña, Homero Urrutia

**Affiliations:** 1 Laboratorio de Biopelículas y Microbiología Ambiental, Centro de Biotecnología, Universidad de Concepción, Victor Lamas 1290, P.O. Box: 160 C, Concepción, Chile; 2 Instituto de Investigaciones Agropecuarias, INIA Remehue. Ruta 5 Norte Km 8-, Osorno, Región de Los Lagos Chile; 3 University of Montpellier, Montpellier, France; 4 Departamento de Ciencias Biológicas, Facultad de Ciencias de la Vida, Universidad Andres Bello, Autopista Concepción Talcahuano # 7100, 4300866 Talcahuano, Chile; 5 Instituto de Investigaciones Agropecuarias, INIA Quilamapu, Región de Ñuble, Chillán, Chile; 6 Departamento de Microbiología, Facultad de Ciencias Biológicas, Universidad de Concepción, Concepción, Chile

**Keywords:** Endophytic actinobacteria, *Streptomyces* sp., Quorum quenching, Confocal laser microscopy, Blackleg, Soft rot, Potato

## Abstract

**Background:**

The native potatoes (*Solanum tuberosum* subsp. *tuberosum* L.) grown in Chile (Chiloé) represent a new, unexplored source of endophytes to find potential biological control agents for the prevention of bacterial diseases, like blackleg and soft rot, in potato crops.

**Result:**

The objective of this study was the selection of endophytic actinobacteria from native potatoes for antagonistic activity against *Pectobacterium carotovorum* subsp. *carotovorum* and *Pectobacterium atrosepticum,* and their potential to suppress tissue maceration symptoms in potato tubers. This potential was determined through the quorum quenching activity using a *Chromobacterium violaceaum* ATCC 12472 Wild type (WT) bioassay and its colonization behavior of the potato plant root system (*S. tuberosum*) by means of the Double labeling of oligonucleotide probes for fluorescence in situ hybridization (DOPE-FISH) targeting technique. The results showed that although *Streptomyces* sp. TP199 and *Streptomyces* sp. A2R31 were able to inhibit the growth of the pathogens, only the *Streptomyces* sp. TP199 isolate inhibited *Pectobacterium* sp. growth and diminished tissue maceration in tubers (*p* ≤ 0.05). *Streptomyces* sp. TP199 had metal-dependent acyl homoserine lactones (AHL) quorum quenching activity in vitro and was able to colonize the root endosphere 10 days after inoculation.

**Conclusions:**

We concluded that native potatoes from southern Chile possess endophyte actinobacteria that are potential agents for the disease management of soft rot and blackleg.

## Background

The world potato (*S. tuberosum*) production in 2018 was 368.2 million tons, with a yield of 209.4 hg ha- 1[1]. One of the greatest threats to productivity is loss caused by infectious bacterial diseases, which, once introduced into the crop, may persist and be disseminated in agricultural environments unnoticed or through latent (asymptomatic) infection of seed tubers [2, 3]. Research has estimated that 60% of these losses are due to rot, produced during the cultivation, transport, and storage of the tubers [4].

*Pectobacterium carotovorum* subsp. *carotovorum* and *Pectobacterium atrosepticum* are the etiological agents that cause blackleg [5] and soft rot (tubers) diseases on potato crops [6, 7]. They may enter the host through natural apertures (lenticels) or wounds and colonize the plant tissue without causing apparent signs or symptoms of the disease until the environmental conditions (free water, anaerobiosis, and temperature) are propitious for the disease development [4, 8]. The virulence of *Pectobacterium* sp. relies on plant cell wall-degrading enzymes (PCWDE), which disrupt the host cell integrity; however, they activate the synthesis of theses exoenzymes when reaching a high population density coordinated by quorum sensing [9] through a complex set of transcription factors and posttranscriptional regulators [10].

There is no efficient chemical control against these diseases, since the ability of bactericidal compounds to disinfect seed tubers is limited [3, 4]. The method to prevent latent infection in the mother tuber is to use seed tubers derived from material not contaminated by *Pectobacterium* sp., which can be obtained by cutting, rapid multiplication in vitro, or by botanical seeds [4, 7, 11, 12]. Biological control is proposed as an alternative and sustainable tool for preventing and controlling infectious diseases in plants [13, 14]. This involves studying the interactions of the controlling agent with the host and the pathogen to reduce the pathogen inoculum or to control the severity of disease symptoms by avoiding the expression of their virulence factors [14, 15].

In potato crops, the rhizospheric bacterial community appears to be dominated by alphaproteobacteria and actinobacteria [16], as does the endophytic bacterial community. However, this may vary according to management systems [17, 18]. On the other hand, different studies have found different isolates of actinobacteria with demonstrated antagonist activity against phytopathogens in vitro and in vivo [9, 19–23]. Facultative endophytes could be crucial to obtain plant probiotic agents because their adaptive behavior to colonize the root surface could be a decisive step for the expression of beneficial effects for the host plant [24–26].

The study of endophytes has emerged as an alternative method to control vascular wilting diseases [27]. Endophytes have been shown to stimulate plant growth [28–30] in addition to inducing biotic stress resistance [31], suppressing disease [32], and effectively competing for the space available for pathogens [13]. The potential of bacterial endophytes to inhibit different fungal and bacterial plant pathogens has been reported [33–35].

There is a unique germplasm of native potatoes in Chile on the island of Chiloé, which is considered a subcenter for the origin of potatoes grown worldwide. The island’s isolation has allowed the proliferation of a variety of native potatoes preserved in small fields, characterized by their shapes, sizes, colors, and phenological characteristics [36]. Currently there is no information on the endophytic microbiota associated with these native varieties; however, according to previous results in different varieties, endophytic actinobacteria is a promising bacterial group for the engineering approaches of the endospheric microbiome [37]. Therefore, we investigated the in vitro antagonist capacity of actinobacteria endophytes present in Chilean native potatoes against *P. carotovorum* subsp. *carotovorum* and *P. atrosepticum* to assess their potential as agents for the preventive disease management of blackleg and soft rot in potato crops.

## Results

### Characterization of endophytic actinobacteria

Ten isolates of putative endophyte actinobacteria were obtained from native Chiloé potatoes differentiated by their phenotypic characterization and 16S ribosomal gene analysis (Table 1, Fig. 3). Of these, nine isolates were related to the genus *Streptomyces* sp*.*, and one isolate was related to the genus *Nocardia* sp. (Table 1, Fig. 3).

**Table 1:** Macro- and microscopic phenotype descriptions of putative endophyte actinobacteria of native Chilean *Solanum tuberosum* subsp. *tuberosum* with their taxonomic identity according to 16S ribosomal gene analysis

Isolate code	Origin of plant tissue	Culture media ^a^			Aerial mycelia^b^	Substrate mycelia^b^	Soluble pigment^c^	Filament diameter	Spore diameter	Spore disposition ^d^	Identity by 16S ribosomal DNA partial gene	Closest taxa strain in NCBI	Nucleotide sequence	Similarity in Blast analysis	Accession in NCBI database
		ISP2	ISP3	ISP4											
H2TP199	Leaf	B	MB	MB	gray	orange	+	0.77 um	0.62 um	Rectiflexibiles	*Streptomyces* sp.	*Streptomyces galilaeus* strain P-YIM40 (GenBank: MT533915.1)	1051 bp	100%	KY242591
G1TP199	Root	MB	MB	B	gray	orange	+	< 0.5 um	< 0.5 um	ND ^d^	*Streptomyces* sp.	*Streptomyces* sp. strain GF22 (GenBank: MN826279.1)	1036 bp	99.2%	KY242592
KR31	Stem	B	B	B	white	brown	+	0.72 um	< 0.5 um	Retinaculiaperti	*Streptomyces* sp.	*Streptomyces* sp. 7R006 (GeneBank: LC497916.1)	803 bp	98.9%	KY242593
DP143	Tubercle	MB	MB	MB	white/gray	brown	+	0.67 um	< 0.5 um	Retinaculiaperti	*Streptomyces* sp.	*Streptomyces* sp. YRA147 (GenBank: JX430828.1)	1104 bp	99.8%	KY242596
A2R31	Stem	MB	EX	B	gray	brown	+	0.77 um	0.89 um	Rectiflexibiles	*Streptomyces achromogenes*	*Streptomyces achromogenes* strain NBRC 12735	969 bp	100%	KY242590
CR34	Tubercle	MB	B	B	ND^e^	cream	–	< 0.5 um	< 0.5 um	ND ^e^	*Nocardia* sp.	*Nocardia* sp. FGT33 (GenBank: KU382293.1)	750 bp	99.9%	KY296348
HP171	Root	MB	MB	EX	white	orange	–	0.67 um	0.66 um	Rectiflexibiles	*Streptomyces* sp.	*Streptomyces galilaeus* strain PSWC-10 (GenBank: MT275786.1)	1027 bp	99.9%	KY242594
MP136	Leaf	MB	MB	MB	black	yellow	–	0.56 um	0.66 um	Spira	*Streptomyces* sp.	*Streptomyces* sp. CH9-7 (GenBank: LC489240.1)	1078 bp	99.8%	KY242595
TP199	Tubercle	EX	B	MB	gray	yellow	–	0.56 um	< 0.5 um	Straight to flexuous chains	*Streptomyces sp.*	*Streptomyces* sp. KK13-6 (GenBank: LC487830.1)	1147 bp	99.6%	KY228978
NP199	Tubercle	MB	MB	MB	gray	yellow	–	0.77 um	0.58 um	ND ^e^	*Streptomyces* sp.	*Streptomyces* sp. HST09 (GenBank: KX130883.1)	1114 bp	99.3%	KY242597

^a^Culture media according to [38], ISP2 (yeast extract and– malt extract agar), ISP3 (oatmeal agar) and ISP4 (inorganic salts and– starch agar). Furthermore, excellent (EX, 76 to 100%), very good (MB, 51 to 75%), good (B, 26 to 50%), poor (P, 1 to 25%), and negative (N, 0%)

^b^Determined on ISP2 medium according to [38]

^c^Negative: -, Positive: +. Determined in ISP1 medium according to [38]

^d^Description according to [39] *e ND* Not determined

**Fig. 1:**
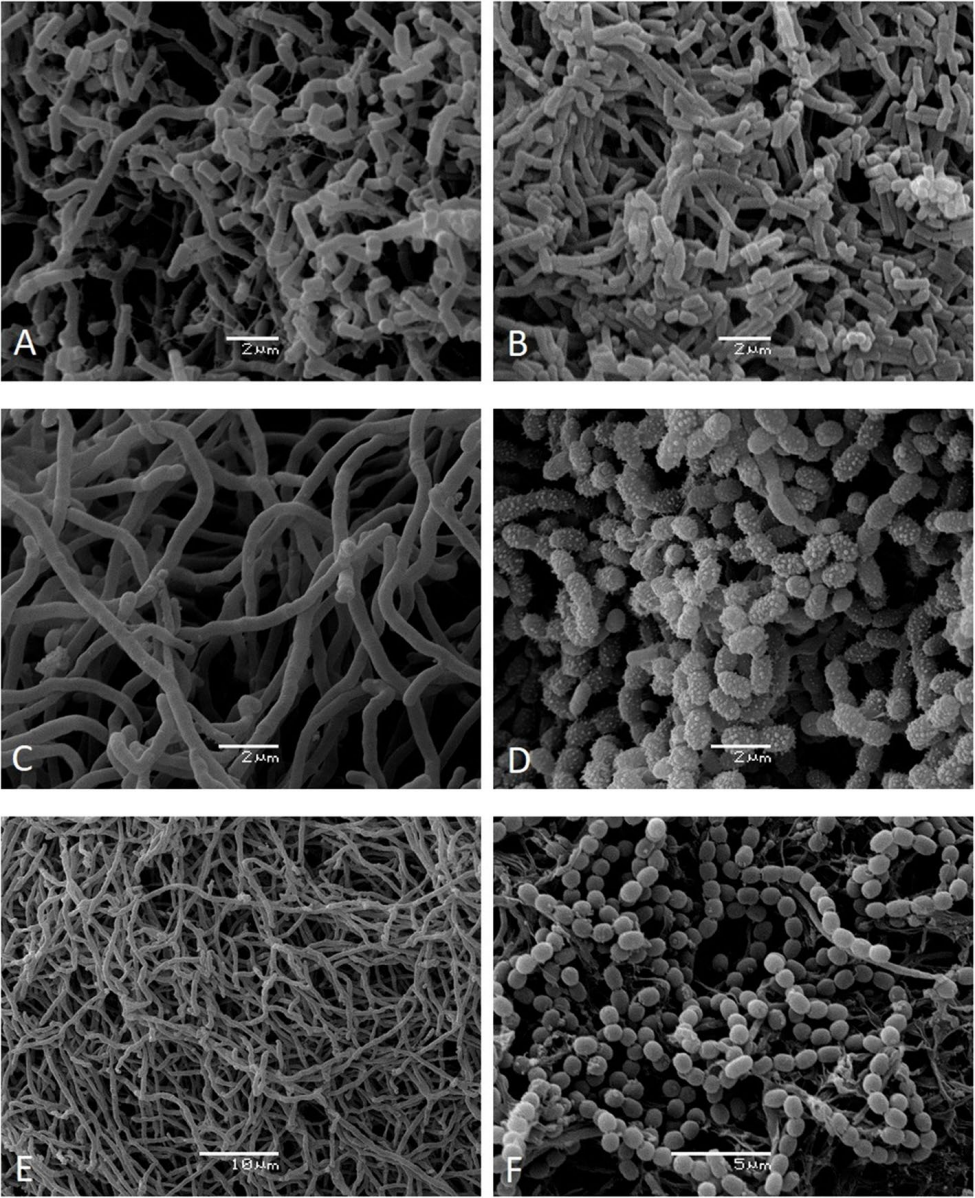
Scanning electron microscopy. **A** *Nocardia* sp. CR34 growth in liquid ISP1 culture after 120 h of incubation at 120 rpm and 28 °C. **B** *Nocardia* sp. CR34 growth in ISP2-agar culture after 14 days of incubation at 28 °C. **C** *Streptomyces* sp. TP199 substrate mycelium growth in liquid ISP1 culture after 120 h of incubation at 120 rpm and 28 °C. **D** *Streptomyces* sp. TP199 aerial mycelium growth in ISP2-agar culture after 14 days of incubation at 28 °C. **E** *Streptomyces* sp. A2R31 substrate mycelium growth in liquid ISP1 culture after 120 h of incubation at 120 rpm and 28 °C. **F** *Streptomyces* sp. A2R31 aerial mycelium growth in ISP2-agar culture after 14 days of incubation at 28 °C. Scale bar: 5 μm

For the following ultramicroscopic visualization using scanning electron microscopy, three bacterial isolated were selected. *Nocardia* sp. CR34 was the only isolation obtained from this genus and was observed according the typical fragment mycelia in liquid (ISP1- tryptone-yeast extract broth) (Fig. 1A) and solid (ISP2- yeast extract and malt extract agar) culture (Fig. 1B).

**Fig. 2:**
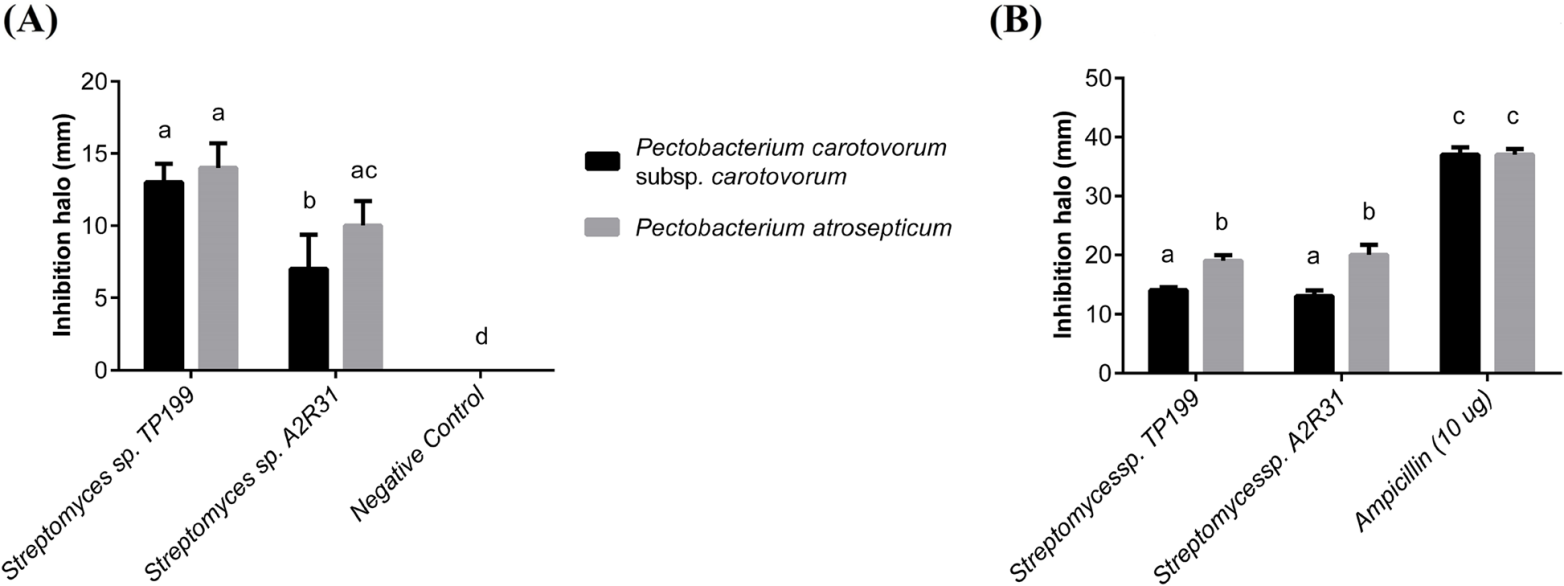
Assessment of the antagonistic activity of *Streptomyces* sp. TP199 and *Streptomyces* sp. A2R31. Isolates against *P. carotovorum* subsp. *carotovorum* and *P. atrosepticum* in vitro. **A** Inhibition growth of pathogens using the cross-streak method, negative control: pathogens without antagonistic; **B** agar plug method, positive control: ampicillin disc (10 μg). Plot mean with SD. Different letters on the top of error bars indicate significative statistical differences (Tukey-test, *p* ≤ 0,05), *n* = 4

Then, *Streptomyces* sp. TP199 and *Streptomyces* sp. A2R31 were selected as they showed antimicrobial activity in vitro (Fig. 2), and their ultramicroscopic visualization showed the long chains of spore growth only in ISP2-agar typical of *Streptomyces* sp. (Fig. 1D and F).

**Fig. 3:**
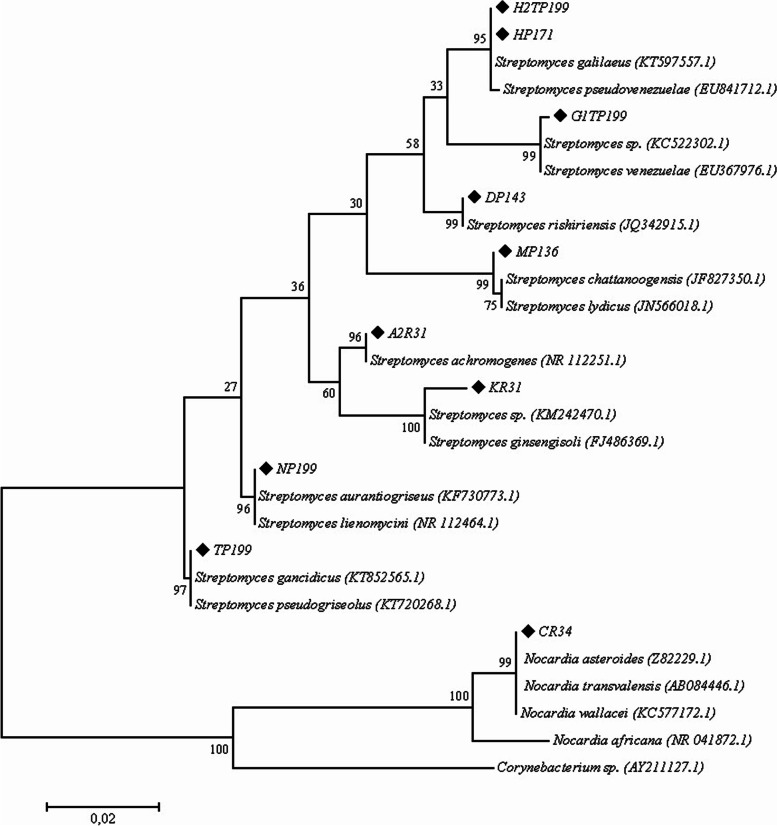
Phylogenetic tree based on an analysis of partial sequences of the 16S ribosomal gene. The maximum likelihood method shows relations between isolates H2T199, HP171, DP143, NP199, TP199, A2R31, KR31, MP136, CR34, and selected members of the *phylum* Actinobacteria from the NCBI database; *Corynebacterium* sp. (AY211127) was used as the tree root. The percentage of replicate trees in which the associated taxa clustered together in the bootstrap test (1000 replicates) are shown next to the branches. The bar indicates 0.02 substitutions per nucleotide

For the *Nocardia* sp. CR34 isolate (Fig. 3), the morphology corresponded to characteristic pseudomycelial growth, both in ISP2-agar culture (Fig. 1B) and in liquid ISP1 culture (Fig. 1A). The other nine isolates were related to *Streptomyces* sp. (Fig. 3), showing mycelial morphology in liquid culture for isolates TP199 (Fig. 1C) and A2R31 (Fig. 1E). The spores could be distinguished on ISP2-agar culture, with a smooth texture in the A2R31 isolate (Fig. 1F) and a warty texture for the TP199 isolate (Fig. 1D).

### Antagonism

Of the ten isolates obtained, only *Streptomyces* sp. TP199 and *Streptomyces* sp. A2R31 inhibited the growth of *P. carotovorum* subsp*. carotovorum* and *P. atrosepticum* in vitro. *Streptomyces* sp. TP199 generated inhibition halos of 13 ± 1.29 mm and 14 ± 1.71 mm against *P. carotovorum* subsp. *carotovorum* and *P. atrosepticum*, respectively, according to the cross-streak method, while *Streptomyces* sp. A2R31 generated inhibition halos of 7 ± 2.37 mm and 10 ± 1.71 mm against the same phytopathogens (Fig. 2A). Using the agar disc diffusion method, *Streptomyces* sp. TP199 generated inhibition halos of 14 ± 0.5 mm and 19 ± 0.96 mm against *P. carotovorum* subsp. *carotovorum* and *P. atrosepticum*, respectively, while the corresponding values for *Streptomyces* sp. A2R31 were 13 ± 0.96 mm and 20 ± 1.71 mm (Fig. 2B). Ampicillin, which was used as the positive control, generated inhibition halos of 37 ± 1.29 mm and 37 ± 0.96 mm against *P. carotovorum* subsp. *carotovorum* and *P. atrosepticum*, respectively (Fig. 2B).

**Fig. 4:**
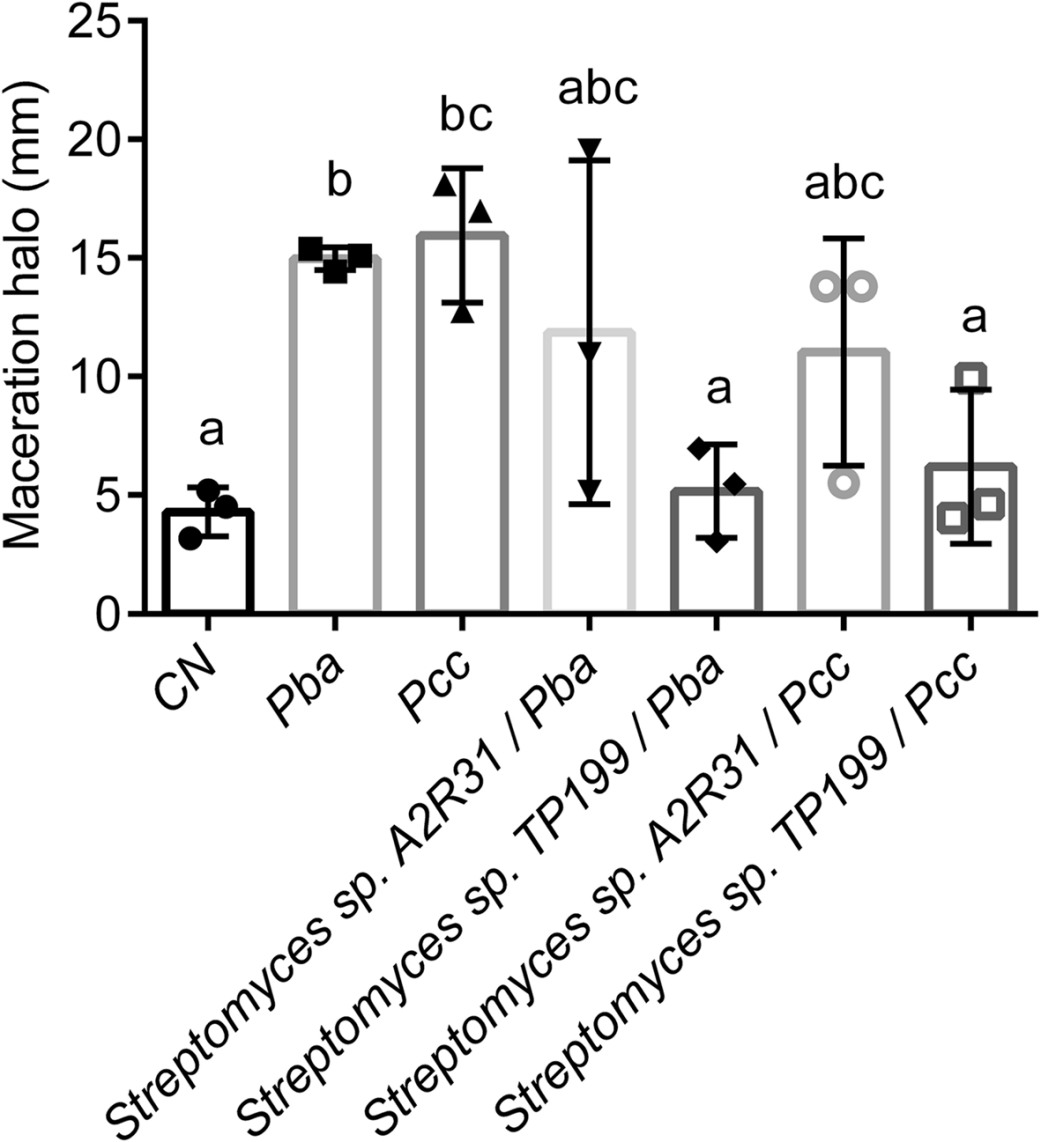
Maceration assay in tuber slices. Maceration tissue halos measured (mm) on tuber slices inoculated with *Pectobacterium carotovorum* subsp. *carotovorum* and *Pectobacterium atrosepticum.* These at a concentration of 10^8^ cells/mL in combination with *Streptomyces* sp. A2R31 and *Streptomyces* sp. TP199 at a concentration of 10^8^ spores/mL, incubated at 28 °C for 72 h; the treatments were: (i) *P. carotovorum* subsp*. carotovorum*, (ii) *P. atrosepticum*, (iii) *Streptomyces* sp. A2R31, (iv) *Streptomyces* sp. (v) *P. carotovorum* subsp*. carotovorum* + *Streptomyces* sp. A2R31 and (vi) *P. carotovorum* subsp. *carotovorum* + *Streptomyces* sp. TP199. Error bars indicate standard deviation of the media. CN: Sterile Nutrient Broth. Pba: *P. atrosepticum*. Pcc: *P. carotovorum* subsp. *carotovorum*. Plot mean with SD. Different letters on the top of error bars indicates statistical significative differences (Tukey-test, p ≤ 0,05), *n* = 3

**Fig. 5:**
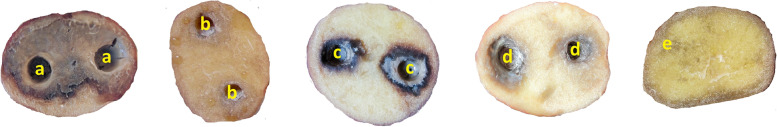
Tuber slice assay. **a**. Wells inoculated with *P. carotovorum* subsp. *carotovorum*. **b**. Wells inoculated with *Streptomyces* sp. TP199. **c**. Wells inoculated with *P. carotovorum* subsp*. carotovorum* + *Streptomyces* sp. TP199. **d**. Wells inoculated with sterile nutrient broth. **e**. Uninoculated slice of the tuber

The effects of *Streptomyces* sp. TP199 and *Streptomyces* sp. A2R31 to inhibit plant tissue maceration were screened by a potato tuber slice assay, which showed, for the control treatments: maceration halos of 4.29 ± 1.03 mm for the well with the sterile nutrient broth, 14.97 ± 0.49 mm for the well inoculated with *P. atrosepticum*, and 15.95 ± 2.83 mm for the well inoculated with the *P. carotovorum* subsp. *carotovorum* alone. On the other hand, when mixing pathogens and antagonists, the inocula resulted in macerated tissue halos of 11.87 ± 7.25 mm for the well inoculated with a mix of *P. atrosepticum* and *Streptomyces* sp. A2R31 in a 1:1 ratio, 5.16 ± 1.97 mm for the well inoculated with a mix of *P. atrosepticum* and *Streptomyces* sp. TP199 in a 1:1 ratio (Fig. 4), 11.03 ± 4.79 mm for the well inoculated with a mix of *P. carotovorum* subsp. *carotovorum* and *Streptomyces* sp. A2R31 in a 1:1 ratio, and 6.19 ± 3.25 mm for the well inoculated with a mix of *P. carotovorum* subsp. *carotovorum* and *Streptomyces* sp. TP199 in a 1:1 ratio (Figs. 4 and 5).

### Quorum sensing inhibition through *Chromobacterium violaceum* ATCC 12472 wild type (WT) bioassay

**Fig. 6:**
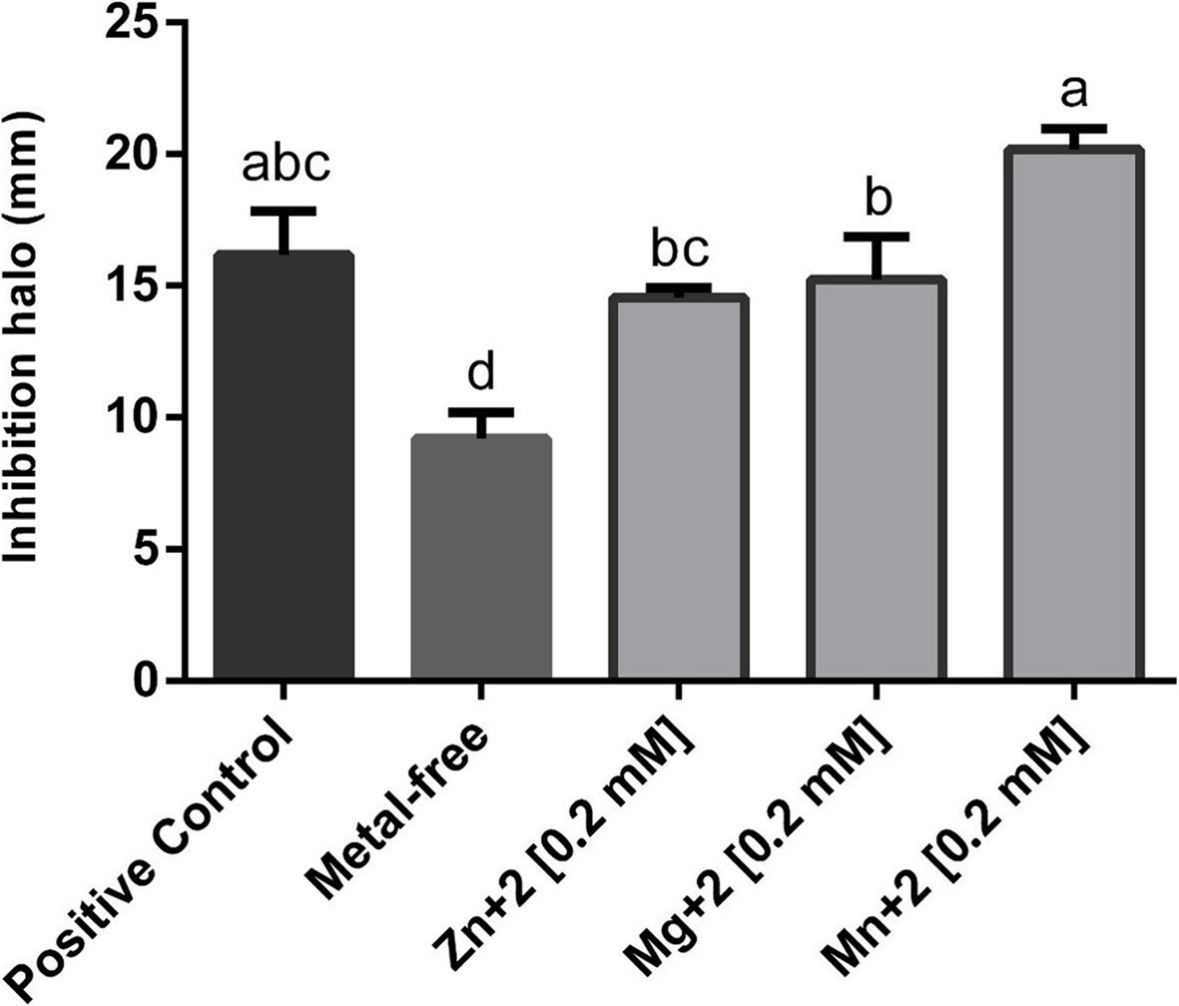
Inhibition of the violacein production of *Chromobacterium violaceum* ATCC 12472 (WT) by culture of *Streptomyces* sp. TP199. This according to the metal used. The bars represent the diameter sizes generated (inhibition halo in mm) of the colorless areas. Plot mean with SD. Different letters on the top of error bars indicate statistical significative differences (Tukey-test, *p* ≤ 0,05), *n* = 3

To complement the inhibitory effect assay of *Streptomyces* sp. TP199 in the decreased tissue maceration in tubers caused by *Pectobacterium* sp., we assessed the interruption of communication signals that regulate the synthesis of pectinolytic enzymes at the transcription level in different strains of *Pectobacterium* sp. For the first experiment, the supernatant of *Streptomyces* sp. TP199 cultures showed no inhibition. Therefore, we proceeded in accordance with [40] and used an AHL (40 μM N-Hexanoyl-DL homoserine lactone (C6-AHL)) solution before performing the *Chromobacterium violaceum* ATCC 12472 (WT) bioassay. On the other hand, studies showed that certain metals are essential for cutting the ester bond in the lactone ring and for proper enzyme folding, and, in the following experiment, *Streptomyces* sp. TP199 was grown in ISP1 supplemented with solutions of three different metals (Mg, Zn, and Mn) at three different final concentrations (0.2, 1, and 2 mM, final). These 12 conditions, defined by the metallic nature and concentration, were tested for *Streptomyces* sp. TP199 from a sporulated culture in ISP2-agar. *Streptomyces* sp. Metal-free TP199, with acyl homoserine lactones (AHL) stimulation showed less inhibition around the control disk (Fig. 6). For each metal used at 1 and 2 mM, no colorless area appeared. All metal concentrations of 0.2 mM in *Streptomyces* sp. TP199 showed an area of inhibition (Fig. 6).

### Endosphere colonization of Streptomyces sp. TP199 in roots potato plants

**Fig. 7:**
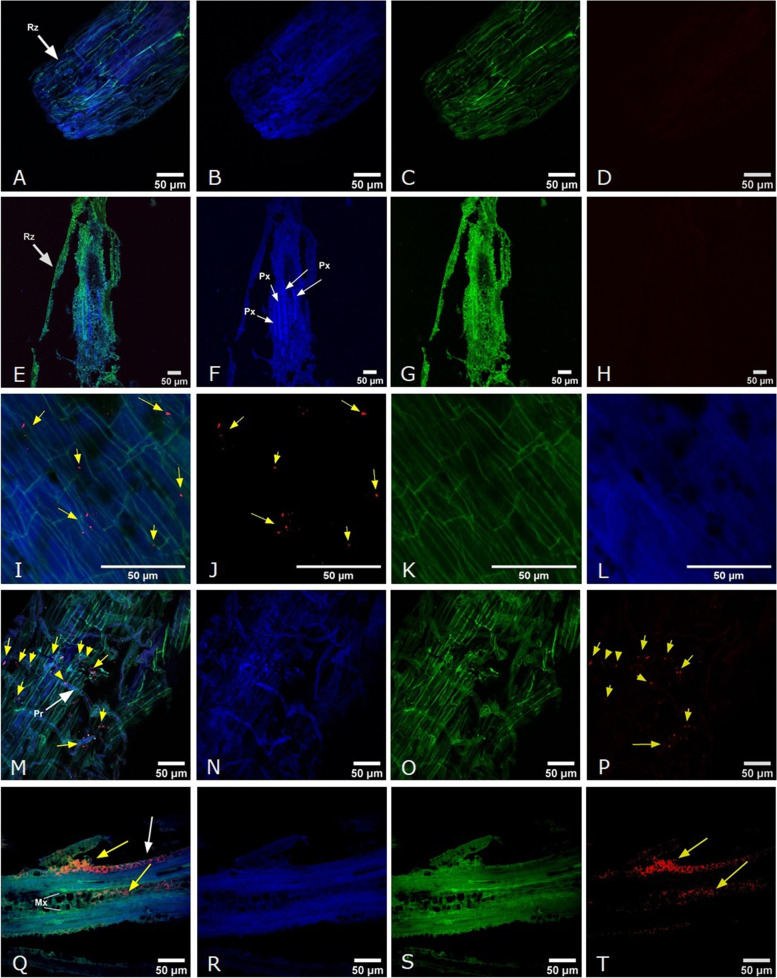
*Streptomyces* sp. TPP199 root colonization patterns targeted by 23S rRNA with Cy5 dye double-labeled HGC69a. Probe visualized by two-photon laser scanning microscopy where autofluorescence of the plant tissue is shown in blue (**B, F, L, N, R**) and green (**C, G, K, O, S**) separately, while the probe is in red separately (**D, H, J, P, T**) and in a merged image (**A, E, I, M, Q**). **A-D** Apical root from a not-inoculated plant, scale bar of 50 μm (day 3 of the assay). **E-H** Longitudinal histological section root from a not-inoculated plant, scale bar of 90 μm (day 10 of the assay). **I-L** Rhizodermis from a 3 day post-inoculated plant, scale bar of 20 μm. **M-P** Root hair growth area from a 3 day post-inoculated plant, scale bar of 60 μm. **Q-T** Longitudinal histological section root from a 10 day post-inoculated plant, scale bar of 60 μm. **A, E, I, M**, and **Q** merged image. **B, F, J, N**, and **R** blue (excitation-emission: 405/410–480 nm). **C, G, K, O**, and **S** green (excitation-emission: 488/490–560 nm) separately, while the 23S rRNA with Cy5 dye double-labeled HGC69a probe is in **D, H, L, P**, and **T** red separately (excitation-emission: 633/638–747 nm). Rz: rhizodermis, Pr: root hair. Mx: metaxylem. Yellow arrows indicate *Streptomyces* sp. TP199 targeted with Cy5 dye double-labeled HGC69a probe fluorescence, and white arrows indicate the plant morphology

Two-photon laser scanning confocal microscopy was used to obtain insights into the *Streptomyces* sp. TP199 root colonization pattern, which was targeted by 23S rRNA with a Cy5 dye double-labeled HGC69a probe (Fig. 7D, H, J, P, T). The identification of the colonization zones in the plant was possible by the autofluorescence of the plant tissue, which is visualized in blue (Fig. 7B, F, L, N, R) and green (Fig. 7C, G, K, O, S). Therefore, in the merged image, the signal from *Streptomyces* sp. TP199 (visualized in red) is contrasted with the autofluorescence of plant tissue (Fig. 7A, E, I, M, Q). The first step of colonization was visualized on a rhizodermal (Fig. 7I) and in the root piliferous zone cells 3 days after inoculation (Fig. 7M). In the case of the histological sections, which correspond to 10 days after inoculation, the greater intensity in the fluorescence corresponding to the HGC69a probe was observed in the area of root hair growth and the metaxylem (Fig. 7Q). The zoom of the vascular bundle’s plant cells allowed us to visualize that the points of greater intensity of the HGC69a probe’s fluorescence were presented in the cell walls and intercellular spaces of eukaryotic cells.

## Discussion

Actinobacteria is a bacterial phylum differentiated by the high content of G + C in its DNA, recognized as one of the main producers of natural bioactive compounds of microbial origin [41] and its biotechnological application prospects [42].

In this study, the presence of facultative endophytic actinobacteria was determined for the first time in native Chilean potatoes by the selective humic acid medium (HV) culture, most of them from the *Streptomyces* genus, which is consistent with the results from cultivation and cultivation-independent population analysis of bacterial endophytes in potato (*S. tuberosum*) [17, 34, 43–47]. According to our results, the morphological and molecular characterization of the isolates obtained was consistent with the genera *Streptomyces*, and not related to pathogenic species or *Nocardia* [39, 48].

Ten isolates of actinobacteria were selected by considering that the disinfection controls presented no growth when cultured. However, this validation is subject to the restrictions governing microorganism culturability [49], as they might be epiphytes resistant to the disinfection process [50, 51]. Thus, the isolates reported here should be considered facultative endophytes, with the exception of *Streptomyces* sp. TP199, which was validated as an endophyte by microscopy [30].

The in vitro results for antagonism (cross streak and diffusion with agar discs) resulted in the selection of *Streptomyces* sp. A2R31 and *Streptomyces* sp. TP199 as antagonists of *Pectobacterium carotovorum* subsp. *carotovorum* and *Pectobacterium atrosepticum*. We observed that only the *Streptomyces* sp. TP199 isolate produced a significant reduction in the tuber tissue maceration assay relative to inoculation with the two phytopathogens alone. This phenomenon may, therefore, be linked to the relative inoculation times of the antagonist and the pathogen since according to [52, 53], a *Streptomyces* sp. isolate was able to reduce the severity of rot symptoms in plants and tubers by 65 to 95% when it was inoculated 24 h before the pathogens. Moreover, the nine facultative actinobacteria endophytes reported in this study without antimicrobial activity could be studied for another plant–microbial interaction.

Isolates of *Streptomyces* were previously reported as antagonists of *Pectobacterium* through an in vitro assessment of anti-microbial activity; for example, the authors in [53] reported four isolates of *Streptomyces* that inhibited the growth of six different strains of *Pectobacterium* (strains B21, K6, M2, Kh6, CFBP5890, and CFBP5889) with inhibition halos in the range of 11 to 27.33 mm, comparable to the results obtained in this work. A further 13 isolates of actinobacteria with antagonistic activity against *Erwinia chrysanthemi* 3937VIII, a phytopathogen that causes soft rot, were obtained; the study also presented notable results from an isolate of *Streptomyces cinereoruber* evaluated as a biocontroller [54].

The synthesis of antimicrobial compounds produced by *Streptomyces* sp. TP199 and *Streptomyces* sp. A2R31 could be induced by the presence of the pathogen in the medium, and this could be the reason why no antimicrobial activity against *P. carotovorum* subsp. *carotovorum* and *Pectobacterium atrosepticum* was observed in the supernatants of the actinobacterial cultures, since the induction of secondary metabolites was reported under the conditions of microbial co-cultures [55, 56]. To test this hypothesis and distinguish a bactericidal effect, *Streptomyces* sp. TP199 and *P. carotovorum* subsp. *carotovorum* was co-cultivated, and no inhibition was observed in the *P. carotovorum* subsp. *carotovorum* growth, according to the live and dead cells count recording and Gompertz model analysis.

Then, to explain the lack of bactericidal effect on *P. carotovorum* subsp. *carotovorum* growth and an effective maceration tissue control in the potato slices assay, we considered a direct effect on the virulence of the pathogen, as shown by [40, 57–60], who assessed the interruption of communication signals that regulated the synthesis of pectinolytic enzymes at the transcription level in different strains of *Pectobacterium* sp. Consequently, the inhibition of violacein synthesis through the *Chromobacterium violaceum* ATCC 12472 (WT) bioassay plates were evaluated to test the AHL inhibition mechanism in *Streptomyces* sp. TP199. First, no inhibition was observed when the supernatants were taken; every 12 h of *Streptomyces* sp. TP199 growth was tested; however, using the research of [61], we reviewed that some AHL-lactonases need a metal in order to degrade the AHL in an optimum way. Different metals were tested, in this experiment, in addition to AHL including: manganese, calcium, magnesium, and zinc. The choice of these metals was made according to their frequency in different strains, in the AHL degradation [61]. Through the results in *C. violaceum* plates, violacein inhibition appeared only for 0.2 mM for each metal, which is a promising result for the inhibition quorum sensing mechanism possibly related to an AHL degrading enzyme metal-dependent and not currently defined for *Streptomyces* sp. species [59, 60].

Finally, this taxa has been described as part of the plant microbiota, colonizing different compartments, such as the endosphere, phyllosphere, and rhizosphere [28, 62]. To determine the colonization capacity of *Streptomyces* sp. TP199 in a commercial potato crop, like an endophyte, it was inoculated in the root system of potato cv. Pukará-INIA plants, which were obtained by micropropagation to obtain plant material free of pathogens. Bearing in mind that the combination of the use of DOPE-FISH with the use of confocal laser microscopy (CLSM) facilitated the exploration of micro-habitats and allowed the observation of microorganisms associated with their host in situ with excellent results, these techniques were used and resulted in the visualization of *Streptomyces* targeted with a double-labeled Cyt5 dye HGC69a probe in the piliferous zone and into the xylem vessels 10 days post-inoculation, similar to the results of [63]; however, it is necessary to make a distinction regarding the passive or active mechanism and to determine if this could confer protection to the plants against *Pectobacterium* sp. infection [62, 64].

To understand this behavior, future studies must determine the chemical nature, concentrations, and diffusion properties of the assemblage of active molecules secreted by the antagonists, interpret more precisely the potential of *Streptomyces* sp. TP199 against *P. carotovorum* subsp. *carotovorum* and *P. atrosepticum,* and validate the mechanism related to blocking the virulence expression of *Pectobacterium* sp. inside the plant and in the tubers [14, 59].

## Conclusions

In conclusion, two isolates of endophytic actinobacteria from native Chilean potatoes (*Solanum tuberosum* L.), *Streptomyces* sp. TP911 and *Streptomyces* sp. A2R31, were found to possess antagonistic activity in vitro against *Pectobacterium carotovorum* subsp. *carotovorum* and *Pectobacterium atrosepticum*; however, only *Streptomyces* sp. TP199 decreased the tuber tissue maceration (approximately 38 and 34%, respectively), and responded to the presence of antimicrobial compounds and AHL signal interference with metal-dependent enzymatic action. *Streptomyces* sp. TP199 also acted as a vegetable probiotic by being able to colonize the root endosphere of potato plants of cv. Pukará-INIA. This suggests that the native Chilean potato possesses endophytic actinobacteria that could be potential agents for the management of the diseases of blackleg and soft rot in potato crops.

## Methods

### Isolation of endophyte actinobacteria

Tubers of native potato (*S. tuberosum* subsp. *tuberosum)* obtained from Chiloé (Southern Chile) were made available by the National Agricultural Research Institute (INIA) Remehue, Osorno (accession number Ch. P11). This institution made the formal identification of the plant material used. A voucher specimen of this material has been deposited in a publicly available herbarium of INIA Remehue (Deposition number not available). They were sown in pots containing sterile trumao soil (Soil derived from frequent volcanic ash in southern Chile) and kept in a greenhouse for 5 months at INIA Quilamapu, Chillán (VIII Region of Chile) to collect plant tissue. Leaves, stems, roots, and tubers were collected from plants that presented no visible disease symptoms in the greenhouse. After collection, the tissues were washed in a soapy solution, and then 2 to 3 g of each tissue was resuspended in phosphate-buffered saline (PBS) solution with Tween-20 (0.01%) and sonicated at 60 Hz for 3 min [65, 66]. The tissues were then immersed in ethanol 70% for 1 min, sodium hypochlorite (NaOCl) at 3% for 4 min, washed three times in sterile water, and finally dried on sterile absorbent paper under a laminar airflow hood. To obtain only endophytes from the phylum Actinobacteria, slices of approx. Four square millimeter were placed on humic acid medium (HV) [67] and supplemented with nystatin (0.05 g/mL) and cycloheximide (0.05 g/mL), designed for the specific growth actinobacteria. They were incubated at 30 °C for 30 days [66, 68]. The surface disinfection of the tissues was validated in HV agar incubated at 28 °C for at least 30 days [66, 68].

### Characterization of the actinobacteria

Macroscopic morphological characterization was conducted out according to the guidelines of [38] in three different culture media: ISP2 (yeast extract and malt extract agar), ISP3 (oatmeal agar) and ISP4 (inorganic salts and starch agar). Growth was categorized by the growth area on a Petri dish as a function of the total area and expressed as a percentage in the following categories: excellent (EX, 76 to 100%), very good (MB, 51 to 75%), good (B, 26 to 50%), poor (P, 1 to 25%), and negative (N, 0%). Measurements were taken using the ImageJ program [69]. The isolates were incubated at 28 °C and observed after 21 days. The production of soluble pigments, the color of the aerial mycelium, and the substrate mycelium color were recorded [39, 48]. Cultures were obtained using the slide technique and incubated for 14 days with ISP2 as the growth medium, then observed under the microscope.

The coverslips with growth were removed and placed on another slide fixed with absolute methanol for 15 min and stained with crystal violet for 1 min. After rinsing and drying, the coverslip was examined under an optical microscope. In each case, the filaments and spore morphology was observed, and the diameter was measured [39, 48]. Isolates representing different taxonomic genera were selected for the examination of cell morphology by scanning electron microscopy [39], both as solid cultures on ISP2 after 14 days of incubation at 28 °C and as liquid cultures in ISP1 (tryptone-yeast extract broth) after 120 h of incubation with shaking at 120 rpm and 28 °C. The products were preserved in 4% glutaraldehyde at 5 °C and processed with scanning electron microscopy at the Microscopy Centre of the University of Concepción, Concepción, Chile.

The taxonomy was determined via 16S ribosomal genes by cultivating the actinobacterial isolates in ISP1 for 120 h at 28 °C and inoculating them to a final concentration of 2.5 × 10^8^ spores/mL. The samples were centrifuged at 12,000 rpm and 4 °C, and the DNA was extracted from the pellets using the ZR Soil Microbe DNA MiniPrepTM kit following the manufacturer’s instructions. For isolates that did not form spores, DNA was extracted from an even covering on ISP2 medium [38]. The 16S ribosomal gene was amplified with the universal primers for bacteria: 9-27F (5′-GAGTTTGATCCTGGCTCAG-3′) and 1541R (5′-AAGGAGGTGATCCAACC-3′) [70]. The PCR product was viewed by electrophoresis in a 1.2% (w/v) agarose gel with ethidium bromide (10 mg/mL) in a transilluminator with UV light [71]. Sequencing services were provided by Macrogen Korea (http://www.macrogen.com/). The rDNA 16S sequences were analyzed using the BLASTn tool (http://blast.ncbi.nlm.nih.gov/Blast.cgi). The sequences produced by Blast were selected with > 98% identity.

MEGA7 software was used to construct the phylogenetic tree [72] using the maximum likelihood method based on the model of [73]. The tree was validated statistically by bootstrapping with 1000 iterations [74], and the initial tree for the heuristic search was obtained by applying the Neighbor Joining method to an estimated distance matrix using the compound maximum probability approach.

### Antibacterial activity

The strains of *Pectobacterium carotovorum* subsp. *carotovorum* and *Pectobacterium atrosepticum* were supplied by the Phytopathology Laboratory of INIA CRI Remehue, Osorno, Chile. The antagonistic activity of the actinobacterial isolates was evaluated using the cross streak agar diffusion technique and agar discs [53, 54]. The cross streak agar procedure is a simple way to test antagonistic properties of actinobacteria through an easy and a rapid semi-quantitative screening, where the inhibition halos could be measure, then let establish a spectrum of inhibiting properties of actinobacteria [75]. The *Pectobacterium* strains were used in the exponential phase (10^8^ cell/mL) cultured in nutrient broth under shaking at 110 rpm and 28 °C for 18 h. For cross streak evaluation, the actinobacterial isolates were sown in a strip across the diameter of the dish on nutrient agar and incubated at 28 °C until sporulation. Then, the *Pectobacterium* was sown in a strip perpendicular to the actinobacteria and incubated for 48 h at 28 °C to observe the formation of the inhibition halos. For evaluation on agar discs, *Pectobacterium* was sown evenly on nutrient agar and then placed on an agar disc (6 mm) with sporulated actinobacteria on ISP2, as well as an agar disc with sterile ISP2 as a blank and a disc with ampicillin (10 μg) as a positive control. These were incubated at 28 °C for 48 h to observe the formation of inhibition halos. Both assays were performed in triplicate for each isolate, and the measured halos were analyzed by means of Tukey HSD test [76].

### Maceration assay in tuber slices

Tubers of Pukará-INIA potatoes were washed with a soapy solution and resuspended in PBS with Tween-20 [0.01%], then sonicated at 60 Hz for 10 min. The tubers were disinfected in sequence in 3% sodium hypochlorite (NaOCl) for 4 min and 70% ethanol for 1 min and flamed. Then, the slices were placed in a laminar flow chamber (for drying). Slices 7 to 8 mm thick were cut and placed in Petri dishes with sterile absorbent paper, and three wells (6 mm) were made in each slice [77–79]. A pathogenic suspension of each strain of *Pectobacterium* (10^8^ cells/mL) was mixed with a suspension of actinobacteria; spores (10^8^ spores/mL) at a 1:1 ratio. The control suspensions were prepared in the same way but with only the pathogen or only the antagonist, mixing the strains with sterile PBS at a 1:1 ratio. A volume of 30 μL of each inoculum was added to each well, and the absorbent paper under the tuber slices was moistened with sterile water. The sterile nutrient broth was used as a negative control. The dishes were then incubated at 28 °C for 72 h, and the macerated tissue diameter around the wells was measured. Each assay was performed in triplicate [77–79], and the mean diameter per treatment was compared using Tukey HSD test (*p* ≤ 0.05 [76];).

### Metals tests and AHL- inhibition assays

Bioassay plates were made with LB-Agar 1,5% medium, covered by a solution of *Chromobacterium violaceum* ATCC 12472 (WT) at 10^7^ cells/mL from the inoculum, in Soft Luria Bertani (LB)-Agar 0,7% [80]. In the first experiment, filter discs were filled with 20 μL of each sampling time supernatant (every 12 h of *Streptomyces* sp. TP199 for 7 days) previously passed through 0,22 μm filters to eliminate the possible presence of bacteria. Two serial dilutions at ½ and ¼ were made from these supernatants [57, 58]. In the second experiments, *Streptomyces* sp. TP199 were grown on ISP2 agar culture for 3 days at 28 °C and then subcultured in ISP1 medium for 1 day. An inoculum of 10^8^ cells/mL of this strain was made. One hundred fifty microliter of both cultures were further incubated in two 96 well microplates at 28 °C with 120 rpm shaking for 3 days.

The metals solutions were adjusted at pH = 7 (MnCl_2_, Zn^2+^, and MgSO_4_) and added at final concentrations of 0,2, 0,5, 1, and 2 mM, according to the experiment. One triplicate of wells did not receive a metal solution. Negative controls were made with filling two triplicates of wells, respectively, with metal solutions only and ISP1 medium. Forty microliters of each well were mixed with an equal volume of a 40 μM N-Hexanoyl-DL homoserine lactone (C6-AHL) [40]. The mixture was incubated at 28 °C with a 120 rpm shaking during 2 h and the reaction was stopped by heating at 95 °C during 10 min. Negative controls were made with the LB liquid medium and positive controls with vanillin (0,01 g/mL), previously proven to have AHL-inhibition in the laboratory. All plates were incubated 24 h at 30 °C to allow the purple development. An inhibition halo of the purple pigment was observed when AHLs production by *C. violaceum* ATCC 12472 (WT) was inhibited. We measured and recorded the results. The mean diameter per treatment was compared using Tukey HSD test (*p* ≤ 0.05 [76];).

### Plant inoculation

Twenty plants were obtained by micropropagation and acclimated in a sterile sand substrate under controlled environmental conditions. The photoperiod was maintained with 16 h light and 8 h dark, 70% relative humidity (R.H.), and 20 ± 2 °C for approximately 3 weeks, until vigorous root growth was observed [81]. Then, 10 plants were selected to control treatment, and 10 plants were inoculated with 10^8^ spores/mL of *Streptomyces* sp. TP199. The root system was inoculated by the dipping method for 10 min, keeping the spores’ movements softly. Then, they were transplanted to independent pots in the sterile substrate, and the conditions of controlled growth were maintained.

Root tissue samples were collected from the control and inoculated plants on days 3 and 10 post-inoculation and were fixed in 4% paraformaldehyde for 24 h and subsequently stored in PBS/EtOH 95.5° (1: 1) at − 20 °C until processing. The HM525 cryostat was used to obtain 30 μm thick cross-sections, which were placed on microscope slides that were previously treated with polylysine (1 mg/mL).

### Double labeling of oligonucleotide probes for fluorescence in situ hybridization (DOPE-FISH)

The probe HGC69a (5′-TATAGTTACCACCGCCGT-3′ )[82] was used for the 16S rRNA gene whose specificity was verified in the Probe Check database (http://microbial-ecology.net/) and was labeled with fluorophore Cy5 at the 5 ‘and 3’ ends of the nucleotide sequence (emission and excitation outside the autofluorescence range of the plant tissue )[83]. Whole root samples and histological root sections on microscope slides (the edge was drawn with hydrophobic pencil) were processed and were pre-treated with achromopeptidase (1 mg/mL) at 37 °C for 15 min and then serially dehydrated with ethanol (50–99.9%, 30 min each stage). A first hybridization step was performed (0.02 mM Tris-HCl, 0.01% SDS, 0.9 M NaCl, and 25% formamide), the probe was added to a final concentration of 15 ng/μL in the dark, and then the samples were incubated at 48 ± 1 °C for 3 h. Subsequently, the samples were drained in a washing solution (0.02 mM Tris-HCl, 0.01% SDS, and 5 M NaCl), preheated to 51 °C, and incubated in the same solution at 51 °C for 20 min. Finally, the samples were dried in the dark in the laminar flow chamber, the immersion solution was added to maintain fluorescence (Prolong, Sigma), and the coverslip was placed.

### Visualization by two-photon confocal laser microscopy

A two-photon confocal laser microscope LSM780 NL0 Zeiss (Advanced Microscopy Center, CMA BIO-BIO, CONICYT PIA ECM-12 Project) linked to the ZEN blue software was used for the visualization and image capture from the treated root samples. Different lasers were used for different ranges of excitation and emission, corresponding to the excitation/emission spectra: 488/490–560 nm, 405/410–480 nm (UV light), and 633/638–747 nm for the probe (far-red light). The Image J program [69] was used to process the images.

## Data Availability

The sequencing data generated in this study are submitted to the Gene-Bank Database (https://www.ncbi.nlm.nih.gov/nuccore, accessions numbers: KY242591, KY242592, KY242593, KY242596, KY242590, KY296348, KY242594, KY242595, KY228978 and KY242597). *Solanum tuberosum* Pukará-INIA is available according to the number 1-20-09-2016-G0-128 of the Registry of Varieties Suitable for Certification of Chile, (https://www.sag.gob.cl/). *Solanum tuberosum* (native from the Chiloe region, Chile) was obtained from the Chilean germplasm network belonging to the National Institute of Agricultural Research (https://web.inia.cl/blog/tag/red-de-bancos-de-germoplasma/). Accession number Ch. P11_9.

## Abbreviations

ATCC: American Type Culture Collection
WT: Wild type
DOPE-FISH: Double labeling of oligonucleotide probes for fluorescence in situ hybridization
AHL: Acyl homoserine lactones
PCWDE: Pectobacteria relies on plant cell wall-degrading enzymes
ISP1: Tryptone-yeast extract broth
ISP2: Yeast extract and– malt extract agar
ISP3: Oatmeal agar
ISP4: Inorganic salts and– starch agar
EX: Excellent
MB: Very good
B: Good
P: Poor
N: Negative
NCBI: *National Center for Biotechnology Information*
ND: Not determined
Pcc: *P. carotovorum* subsp*. carotovorum*
Pba: *P. atrosepticum*
23S rRNA: 23S ribosomal RNA
HV: Humic acid medium
INIA: National Agricultural Research Institute
PBS: Phosphate-buffered saline
HSD: Honestly significant difference
RH: Relative humidity
SDS: Sodium dodecyl sulfate
UV: Ultraviolet
